# Early and consistent overexpression of *ADRM1* in ovarian high-grade serous carcinoma

**DOI:** 10.1186/s13048-017-0347-y

**Published:** 2017-08-07

**Authors:** Rosie T. Jiang, Anna Yemelyanova, Deyin Xing, Ravi K. Anchoori, Jun Hamazaki, Shigeo Murata, Jeffrey D. Seidman, Tian-Li Wang, Richard B. S. Roden

**Affiliations:** 1 Department of Pathology, The Johns Hopkins University, Baltimore, MD 21231 USA; 2 Department of Oncology, The Johns Hopkins University, Baltimore, MD 21231 USA; 3 Departments of Gynecology and Obstetrics, The Johns Hopkins University, Baltimore, MD 21231 USA; 4 Division of Gynecologic Pathology, The Johns Hopkins University, Baltimore, MD 21231 USA; 5 Laboratory of Protein Metabolism, The University of Tokyo, Bunkyo-ku, Tokyo, Japan; 6 Division of Molecular Genetics and Pathology, Center for Devices and Radiological Health, Food and Drug Administration, Silver Spring, MD 20993 USA

**Keywords:** ADRM1, RPN13, STIC, Proteasome inhibitor, Ovarian/fallopian tube cancer

## Abstract

**Background:**

Ovarian carcinoma is highly dependent on the ubiquitin proteasome system (UPS), but its clinical response to treatment with the proteasome inhibitor bortezomib has been disappointing. This has driven exploration of alternate approaches to target the UPS in ovarian cancer. Recently, proteasome inhibitors targeting the 19S regulatory particle-associated RPN13 protein have been described, such as RA190. RPN13, which is encoded by *ADRM1*, facilitates the recognition by the proteasome of its polyubiquinated substrates. Inhibition of RPN13 produces a rapid, toxic accumulation of polyubiquitinated proteins in ovarian and other cancer cells, triggering apoptosis.

Here, we sought to determine if RPN13 is available as a target in precursors of ovarian/fallopian tube cancer as well as all advanced cases, and the impact of increased *ADRM1* gene copy number on sensitivity of ovarian cancer to RA190.

**Methods:**

*ADRM1* mRNA was quantified by RNAscope in situ hybridization and RPN13 protein detected by immunohistochemistry in high grade serous carcinoma (HGSC) of the ovary and serous tubal intraepithelial carcinoma (STIC). Amplification of ADRM1 and sensitivity to RA190 were determined in ovarian cancer cell lines.

**Results:**

Here, we demonstrate that expression of *ADRM1*mRNA is significantly elevated in STIC and HGSC as compared to normal fallopian tube epithelium. *ADRM1* mRNA and RPN13 were ubiquitously and robustly expressed in ovarian carcinoma tissue and cell lines. No correlation was found between *ADRM1* amplification and sensitivity of ovarian cancer cell lines to RA190, but all were susceptible.

**Conclusions:**

RPN13 can potentially be targeted by RA190 in both in situ and metastatic ovarian carcinoma. Ovarian cancer cell lines are sensitive to RA190 regardless of whether the *ADRM1* gene is amplified.

**Electronic supplementary material:**

The online version of this article (doi:10.1186/s13048-017-0347-y) contains supplementary material, which is available to authorized users.

## Background

Missense mutation of *TP*53 is the dominant driver in ovarian/fallopian tube cancer [1–4]. Indeed *TP53* is mutated in nearly all high grade serous carcinomas (HGSC), the histotype responsible for most deaths from ovarian cancer [5, 6], and is also mutated in its precursor, serous tubal intraepithelial carcinoma (STIC) [7–9], suggesting this is a key and early event in carcinogenesis [10]. In addition to inactivating wild type TP53 function, these mutations frequently confer gain-of -function properties including redirection of Nrf2 to upregulate the proteasome [11]. The aberrant metabolism of ovarian cancer cells produces an excess of misfolded proteins that are polyubiquitinated for targeted degradation via the proteasome, and this is associated with proteasome upregulation [12]. Consequently, ovarian cancer cells are both highly dependent on proteasome function and especially sensitive to treatment with a proteasome inhibitor [12–14]. The FDA-approved drug bortezomib, approved for the treatment of multiple myeloma and mantle cell lymphoma, inhibits 20S core particle (CP) proteolytic function producing a rapid and toxic buildup of denatured protein aggregates [15]. The cells attempt to ameliorate this stress via the unfolded protein response (UPR) which promotes re-folding via chaperone upregulation as well as the rapid sequestration and degradation of the misfolded, ubiqutinated proteins. Inability to relieve proteotoxic stress and achieve homeostasis induces TP53-independent cell death [16]. Indeed, treatment with bortezomib slowed the growth of ES-2 ovarian carcinoma xenograft, although alone it did not cure the mice [12].

Despite this promise, early studies with bortezomib in patients with ovarian cancer or other solid tumors have demonstrated minimal clinical benefit. Initial phase I studies suggested a dose of 1.3 mg/m^2^ in combination with cisplatin/carboplatin, paclitaxel or pegylated liposomal doxorubicin had manageable neurotoxicity [17–22], although in combination with oxaliplatin a maximum dose of 1.0 mg/m^2^ bortezomib was suggested because of neuropathy [23]. However, a phase II study of bortezomib alone a dose of 1.3 mg/m^2^ in platinum-sensitive epithelial or primary peritoneal cancer showed minimal activity as a single agent [17]. Likewise, a phase II study of bortezomib and pegylated liposomal doxorubicin showed the combination was well tolerated, but the antitumor activity was considered insufficient to warrant further investigation [21]. Thus, despite bortezomib’s efficacy against multiple myeloma, its toxicity, and limited activity in solid tumors has driven development of inhibitors of the ubiquitin-proteasome system (UPS) with alternative mechanisms to treat ovarian cancer.

RPN13, which is encoded by the gene *ADRM1*, is a ubiquitin receptor tethered to the 19S regulatory particle (RP) of the proteasome via interaction of its amino terminal Pru domain to RPN2 [24]. The Pru domain of RPN13 and RPN10 cooperate to capture K48-linked polyubiquitin chains bound to proteasome substrates. The carboxy terminal domain of RPN13 recruits and promotes the deubiquitinase activity of UCHL5/UCH37 which removes distal ubiquitin from the polyubiquitin chains linked to these substrates [25–27]. Deubiquitination of the substrate is followed by unfolding and transfer to the 20S CP for its proteasomal degradation.

*ADRM1* was originally identified as ADhesion Regulating Molecule-1 in metastatic tumor cells [28]. Several observations have driven interest in *RPN13*’s role in cancer biology; 1) early studies suggested that *ADRM1* transcript levels commonly upregulated in diverse tumor types [29], 2) *ADRM1* was identified as a recurrent amplification target in ovarian cancer [30–32], colorectal carcinoma [33, 34], gastric cancer [35, 36], 3) knockdown of *ADRM1* transcripts suppressed proliferation, growth in soft agar and migration of diverse cancer lines but not normal cells [31, 34–39], 4) over-expression of RPN13 via ectopic expression of *ADRM1* enhanced proliferation [32, 35], 5) *ADRM1*-encoded RPN13 is required for cell cycle progression [40]. Fejzo et al. also observed that *ADRM1* overexpression is significantly correlated with higher stage, shorter time to recurrence and survival [32]. RNAi knock-down of *ADRM1* transcripts in OAW42 cells triggers apoptosis, as is observed upon treatment with RA190. Conversely, using ectopic over expression of *ADRM1* in ES2 cells to examine its biologic function in ovarian cancer cells, they observe that *ADRM1* over-expression increases cell proliferation, migration and growth in soft agar. From these observations, Fejzo et al. proposed that *ADRM1* is “an oncogene and therapeutic target for ovarian cancer” [32].

Anchoori et al. recently developed a promising anticancer drug, RA190, which covalently binds to RPN13 C88 in its Pru domain [36, 41]. RA190 has promising activity against ovarian cancer, cervical cancer and multiple myeloma (MM) in mouse models by triggering an unresolved unfolded protein response (UPR) and then TP53-independent apoptosis [41]. RPN13 expression is dramatically elevated in clinical samples of MM compared to normal plasma cells [42]. Furthermore, RPN13 knockdown by *ADRM1* siRNA*,* or RA190 treatment causes loss of MM cell viability, including ex vivo patients’ samples and bortezomib-resistant lines, via caspase-dependent and UPR-related apoptosis [42]. Using CRISPR/Cas9 knockout of *ADRM1*, we show RA190 activity is dependent upon RPN13. Finally, RA190 treatment was well tolerated, inhibited MM xenograft tumor growth and extended survival [42]. Recently, another group developed KDT11, a reversible ligand of RPN13, which they found to be selectively toxic for MM cells. KDT11 provides further validation of RPN13 as an anticancer drug target, but does not have properties conducive to in vivo use [43]. Lu et al. identified a 38 aa peptide of RPN2 that binds to RPN13 Pru domain with 12 nM affinity. Overexpression of this peptide in 293T cells prevents RPN13 binding to the proteasome resulting in an increase in the cellular load of poly-ubiquitinated proteins [44].

These observations support RPN13 as a treatment target for ovarian cancer, but it is not clear whether it is expressed in all cases and if *ADRM1* amplification impacts sensitivity to RA190. Recent findings suggest that the precursor lesion for HGSCs, STIC, arise at the fimbriated end of the fallopian tubes and shed cancer cells that implant on the surface of the ovary and peritoneum [45–49]. STIC typically exhibit high proportions of proliferating cells and missense *TP53* mutations [48, 50], but it is not known if they also express RPN13 (and to what level) and thus whether STIC are also potential treatable by RA190.

## Results

### Validation of a highly sensitive and specific Chromogenic In Situ Hybridization (CISH) assay for *ADRM1* transcript levels

To develop a sensitive *ADRM1* mRNA CISH assay, we used a custom hybridization probe for the RNAscope® 2.0 assay from Advanced Cell Diagnostics (ACD) that is designed for detection at the single transcript level. To establish its ability to assess mRNA levels, *ADRM1* levels were measured in 4 HGSC patients with matching frozen tissue samples and formalin-fixed, paraffin embedded (FFPE) tissue blocks by mRNA CISH or qRT-PCR, respectively (Additional file 1: Figure S1A, S1B). Levels of *ADRM1* mRNA measured with qRT-PCR correlated with RNAscope® DAB dots (numbers of the dot-like signals) in RNAscope® demonstrating semi-quantitative nature of RNAscope® 2.0 assay.

### Elevated *ADRM1* mRNA expression in matched STICs and HGSCs

**Fig. 1:**
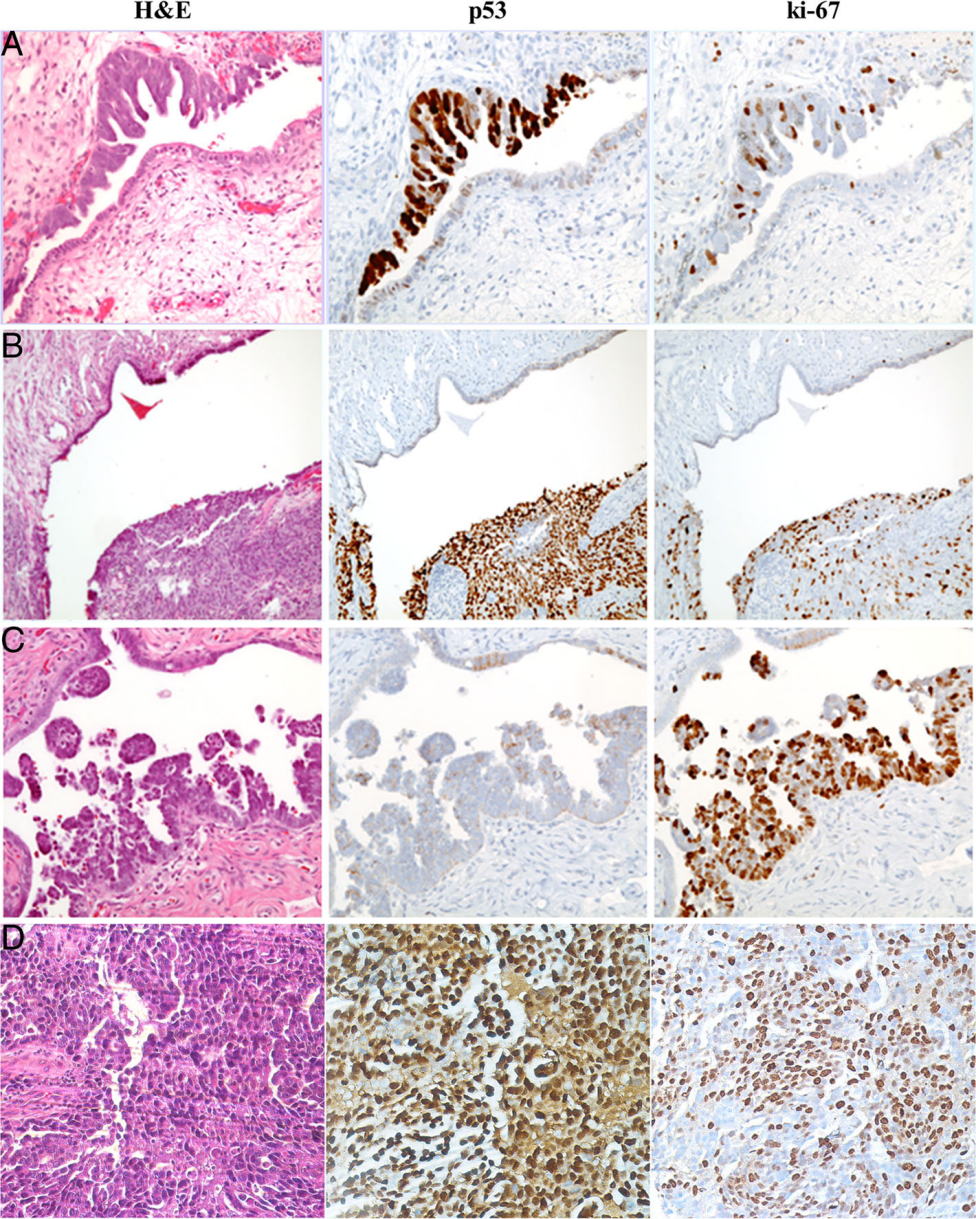
Identification of STICs. Samples from 8 patients with matched STICs and invasive carcinomas were immunostained for TP53, and ki-67. Representative images shown of STICs with matched normal fallopian epithelium (**a**, **b**, **c**) stained with H&E, p53, and ki-67. TMAs containing HGSC samples from 13 patients with 5 tissue cores per patient were also stained with H&E, p53, and ki-67 (representative case shown in **d**)

Formalin-fixed, paraffin embedded blocks of 11 ovarian cancer cases were selected by three gynecologic pathologists who independently assessed the presence of STIC and HGSC carcinoma using H&E, ki-67, and p53 staining to probe morphology, proliferation and, − surrogate of *TP53* muational status, respectively. Of 11 patient samples, 7 FFPE blocks contained matched normal fallopian tube, STIC, and carcinoma (Fig. 1, Rows 1–3). Tumor tissue samples from an additional 13 HGSC patients were arrayed on a TMA containing 5 cores per patient (Table 1, Fig. 1, Row 4).

**Table 1:** TMA patient information

Patient ID	Age	Race	Stage/grade	
Patient 1	47	W	IA	HG
Patient 2	51	W	IIIC	HG
Patient 3	61	W	IIIC	HG
Patient 4	58	B	IIIC	HG
Patient 5	69	B	IIIC	HG
Patient 6	76	W	IIIC	HG
Patient 7	61	W	IIIC	HG
Patient 8	60	B	IIIC	HG
Patient 9	61	W	IV	HG
Patient 10	unknown	unknown	unknown	HG
Patient 11	55	Alaskan	IIIC	HG
Patient 12	58	B	IIIC	HG
Patient 13	75	W	IIIC	HG

Age, race, and stage for 13 patients included in TMA analysis

*W* white, *B* Black, *HG* High-grade, *unk* unknown

**Fig. 2:**
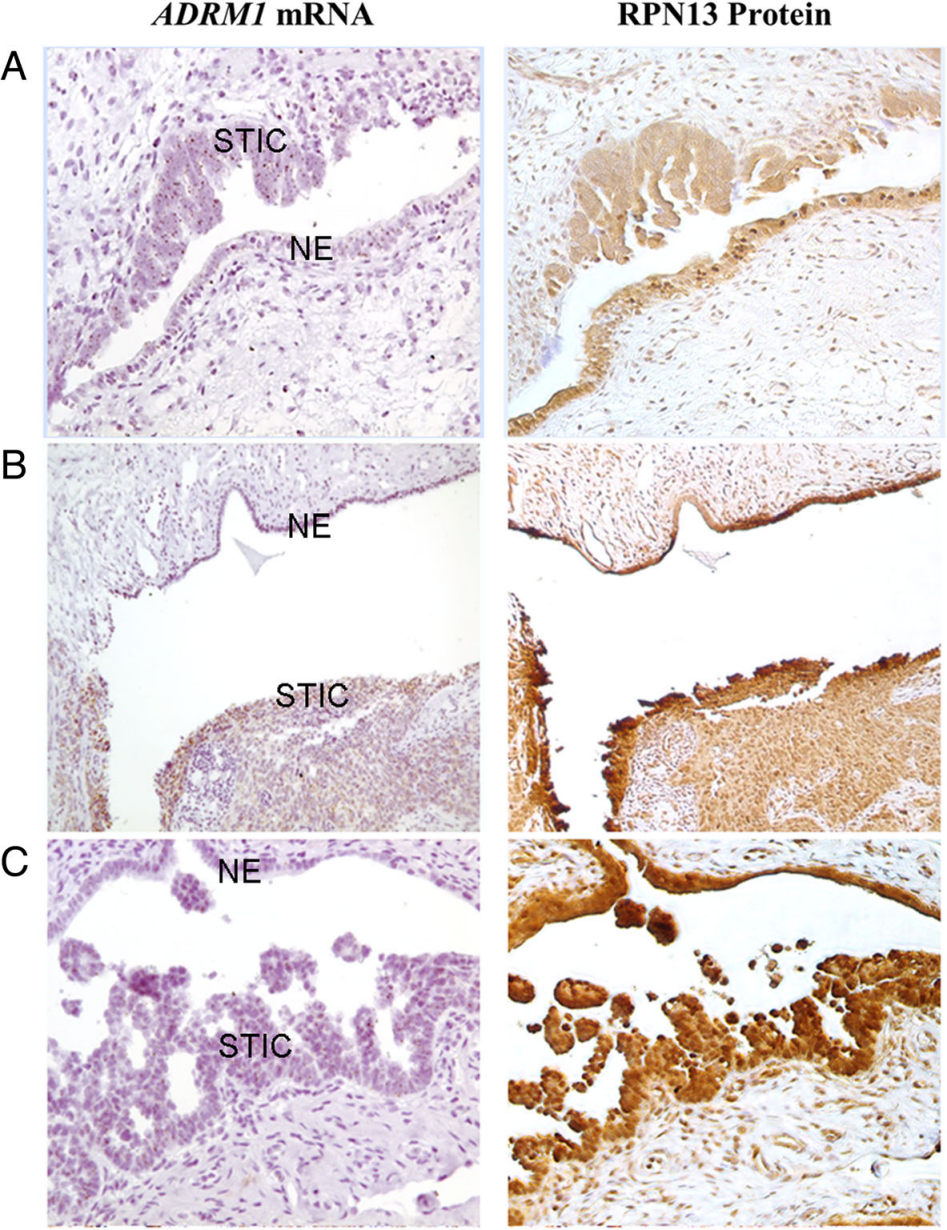
Elevated *ADRM1* mRNA and consistent RPN13 expression in STICs and HGSCs. *ADRM1* mRNA levels in 7 matched normal fallopian tube epithelium, STICs, and HGSCs and 15 TMA HGSCs were probed CISH by RNAscope® 2.0 assay. STICs and matched HGSC show elevated levels of *ADRM1* mRNA as compared to adjacent normal fallopian tube. IHC for RPN13 protein levels in normal fallopian tube epithelium (NE), STICs, and HGSCs show diffuse nuclear and cytoplasmic staining in both normal fallopian tube epithelium and STIC (**a**, **b**, **c** match Fig. 1a–c respectively)

The presence of detectable RNA in the tissue samples was verified by using positive control peptidyl prolyl isomerase B (PPIB) probe (Additional file 2: Figure S2). Next, we utilized the *ADRM1* mRNA CISH assay to probe adjacent slides from the samples of 7 ovarian cancer patients with matched STICs, normal fallopian tube epithelium, and the additional samples from 13 HGSC patients. *ADRM1* mRNA was detected in both normal fallopian tube epithelium as well as STIC (Fig. 2). Expression levels were assessed semi-quantitatively by gynecologic pathologist (DX) as higher in STIC tissue as compared to normal fallopian tube epithelium in all samples (*n* = 7). *ADRM1* mRNA levels in matched invasive carcinoma on same slides were considered to be a similar to those in adjacent STIC. This implies that the elevation of *ADRM1* mRNA levels is an early event that does not obviously increase further as disease progresses.

**Fig. 3:**
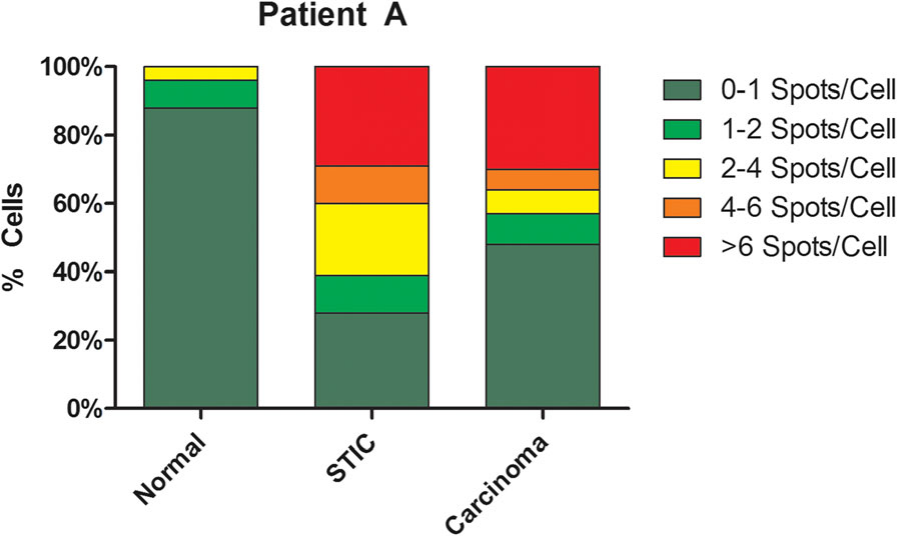
SpotStudio Quantification of RNA-CISH in fallopian tube epithelium, STICs, and HGSCs. Using ACD SpotStudio Software, single cell analysis for CISH were done on all 7 matched normal, STIC, and HGSC samples. STICs and HGSC showed a higher percentage of cells overexpressing *ADRM1* mRNA as compared to fallopian tube epithelium. Distribution of cells containing higher number of *ADRM1* mRNA spots increases FT progresses to STIC and carcinoma. Collectively, STIC and HGSC exhibited significantly different levels as compared to normal (*P* = 0.009, *P* = 0.003, paired T-test). One ﻿representative case (Patient A) is presented, and the remainder (Patients B-G) are presented in Additional file 4: Figure S4

To confirm the semi-qualitative analysis of the gynecologic pathologist, automated quantification was used on the 7 STIC confirmed patients by ACD SpotStudio Software, which allows for direct quantification of target RNA molecules in single cells. After defining regions of interest (ROIs) including normal fallopian tube epithelium, STIC, and invasive high grade serous carcinoma, SpotStudio defines average cell size and quantifies DAB-stained (brown) dots per cell (Additional file 3: Figure S3). Overall, levels of *ADRM1* mRNA were not significantly different between STIC and HGSC (*P* = 0.4) but each was significantly elevated as compared to matched normal epithelium (*P* = 0.009 and *P* = 0.003 respectively, paired T-test). STIC (~50%) and matched HGSC (~33%) showed a higher proportion of cells expressing high levels (>6) of RPN13 mRNA dots per cell (~23%) as compared to normal epithelium (Fig. 3, Additional file 4: Figure S4).

To determine whether RPN13 protein is expressed in these tissues, IHC was performed on adjacent serial sections with mouse monoclonal anti-RPN13 antibody (Fig. 2). As expected for a proteasome protein, RPN13 IHC showed diffuse nuclear and cytoplasmic staining. The specificity of the immunohistochemistry assay was confirmed by comparing the staining of HCT116 wild type cells and an *ADRM1* CRISPR knockout HCT116 clone (Additional file 5: Figure S5). RPN13 expression was detected in STIC and HGSC as well as normal fallopian tube epithelium although there was no clear indication of change in protein level based on this semi-quantitative technique (Fig. 2).

### Consistent expression of RPN13 in HGSC

**Fig. 4:**
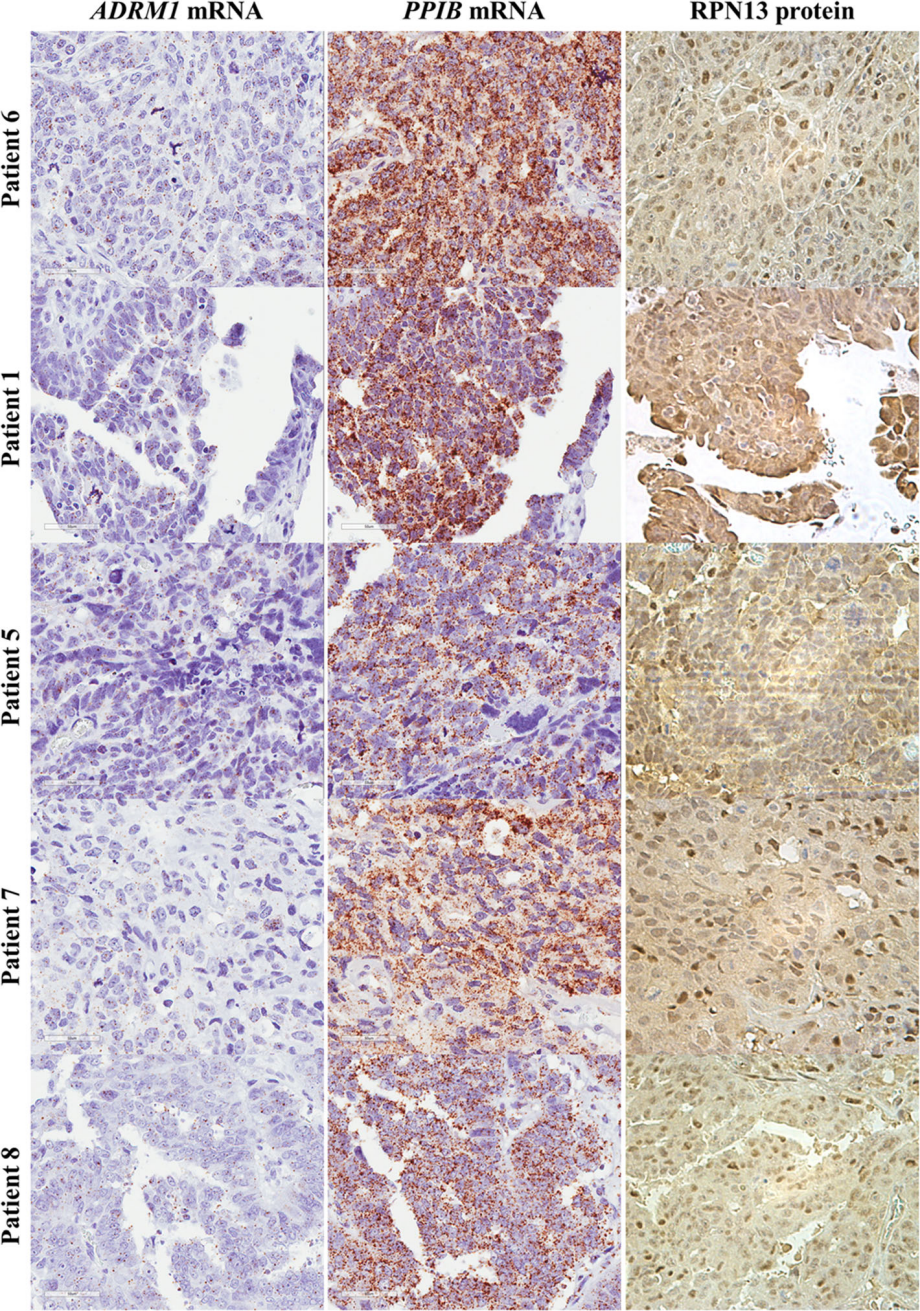
Variation of *ADRM1* mRNA expression in carcinoma. TMA HGSCs showed variability in RNA integrity as exhibited by variable staining with housekeeping mRNA, *PPIB*. However, even compared within similar levels, *ADRM1* mRNA levels varied but were present in all samples. Consistent expression and similar RPN13 levels were found through IHC staining

All cases tested (*n* = 13), regardless of HGSC stage, maintained expression of both *ADRM1* mRNA (determined by in situ hybridization) and RPN13 protein as visualized by immunohistochemistry (Fig. 4). Expression of RPN13 protein was further confirmed by Western blotting of same HGSC samples (*n* = 4) used for RNA-CISH verification (Additional file 1: Figure S1C). When grouped by similar *PPIB* mRNA levels, tissue exhibiting variable *ADRM1* mRNA levels exhibited similar amounts of RPN13 (Additional file 6: Figure S6, Additional file 7: Figure S7).

### Amplification of *ADRM1* does not correlate with expression of RPN13 or sensitivity to RA190

**Fig. 5:**
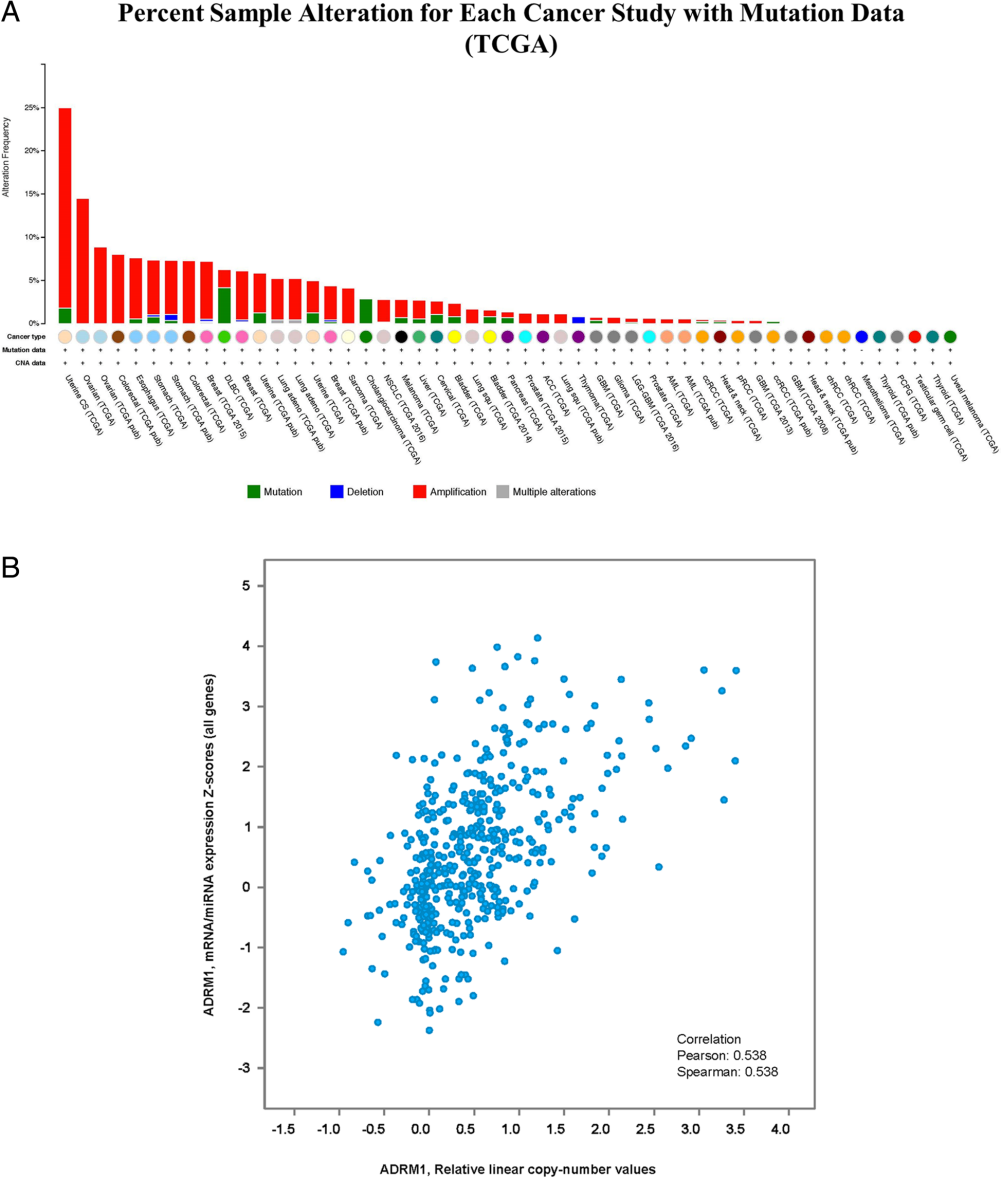
Amplification and expression levels of *ADRM1.* **a** The Cancer Genome Atlas (TCGA) reports that *ADRM1* gene amplifiction in approximately 8–15% of high-grade serous ovarian cancers found across two databases (TCGA, Provisional and Nature 2011) that included 1068 samples in total. [51, 52]. **b** The high-grade serous ovarian cancer TCGA, Nature 2011 database including 489 cases, shows a moderate correlation. of copy number and mRNA levels

**Fig. 6:**
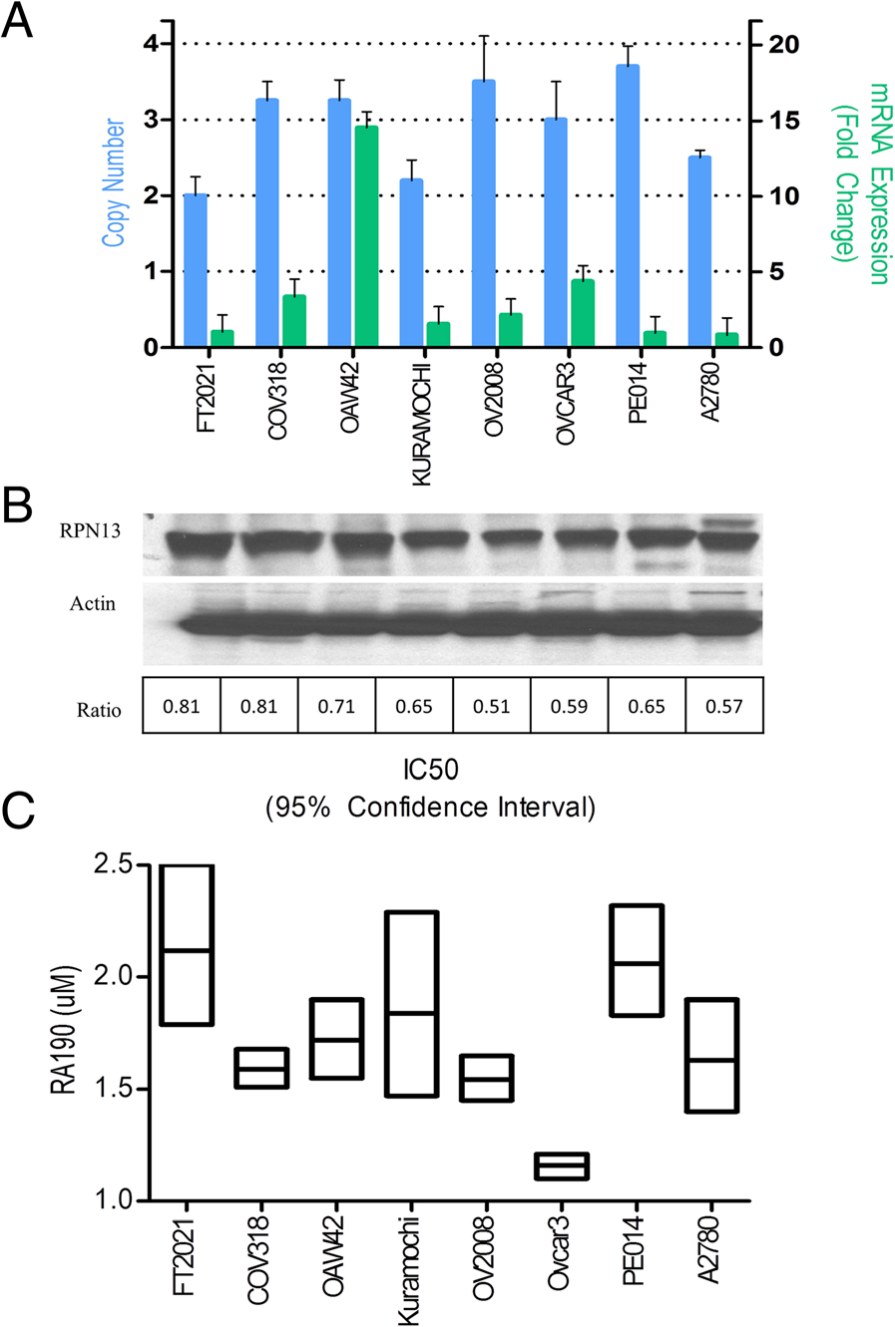
Amplification of RPN13 does not alter expression of protein. **a** Amplification of *ADRM1* was assayed by Taqman Gene Assay and analyzed by CopyCaller™ Software for 7 ovarian cancer and 1 immortalized fallopian tube epithelial (FT2021) cell lines measured. Similarly, purified mRNA was measured by qRT-PCR and found to have no correlation with amplification status. **b** As assayed by Western blot, RPN13 protein levels vary little despite *ADRM1* gene amplification status. Ratio to loading control, ß-actin, was calculated by comparing pixel density as measured by ImageJ. C) XTT cell viability assay of various HGSC cell lines with known RPN13 CNV. All cell lines were shown to be sensitive to RPN13 inhibition by RA190 regardless of amplification status

The Cancer Genome Atlas (TCGA) Research Network collected tumor specimens and analyzed key genomic changes by mRNA-seq, whole exome DNA-seq, miRNA-seq, methylation, copy number, in over 11,000 patient samples encompassing 33 different types of cancer [51, 52]. From analysis of TCGA data it is apparent that ovarian cancer has a higher frequency of *ADRM1* amplification (~8–15%) as compared to other cancers (Fig. 5a) and amplification is moderately correlated with elevated mRNA expression level (Pearson coefficient = 0.538, Fig. 5b). *ADRM1* amplification and mRNA overexpression could lead to differences in sensitivity to RPN13 inhibitors, such as RA190. To test this hypothesis, we established the copy number variation status for a panel of *n* = 7 ovarian cancer cell lines in comparison to the SV40 large T-antigen immortalized normal fallopian tube cell line FT2021 using a TaqMan® Copy Number assay (Fig. 6a, left). Previously TCGA reported cell lines COV318 and OAW42 were amplified. Using the TaqMan® Copy Number assay and determination by the CopyCaller™ Software, this was confirmed. Additional cell lines such as OV2008 and PE014 were also identified by CopyCaller™ Software as amplified. A2780, Kuramochi, and the SV40 large T antigen immortalized normal fallopian tube cell line FT2021 had normal copy number of *ADRM1*, by this approach. However, OVCAR3 was also amplified by CopyCaller™ Software, differing from TGCA results. Among these cell lines, *ADRM1* mRNA levels (Fig. 6a, right) are not correlated to *ADRM1* amplification, although this may reflect the relatively small sample size. Further, the panel of cell lines showed largely similar RPN13 protein levels by Western blot (Fig. 6b) regardless of amplification status, suggesting possible post-transcriptional regulation of RPN13 expression.

To address the hypothesis that variability in *ADRM1* amplification and mRNA expression could potentially alter sensitivity to RA190, XTT cell viability assays were run on HGSC cell lines A2780, Kuramochi, PE014, OVCAR3, OAW42, and COV318, with differing *ADRM1* copy number. All cell lines were sensitive to RA190, with IC50s ranging from 1.2–2.3 μM. There was no apparent correlation between *ADRM1* copy number variation and sensitivity to RA190 in this limited panel of cell lines (Fig. 6c).

## Discussion

The overabundance of misfolded proteins and consequent increased reliance on the ubiquitin proteasome system to relieve proteotoxic stress make ovarian carcinoma an attractive candidate for targeted proteasome inhibition. Recent inhibitors targeting the 19S ubiquitin receptor RPN13 have demonstrated therapeutic potential against cultured cancer cell lines as well as in vivo models and raised questions about RPN13 expression patterns and its functional role in cancer biology [41]. In this study, we have validated a chromogenic in situ hybridization assay to visualize *ADRM1*mRNA levels in normal fallopian tube epithelium, STICs, and HGSCs to determine their relationship to ovarian carcinogenesis. We show that overexpression of *ADRM1* mRNA is an early event found in all probed STIC, the precursor lesions of HGSC. Further, we confirmed expression of RPN13 protein in normal fallopian tube epithelium, STIC and the ovarian tumors. The specificity of *ADRM1* overexpression to developing tumors implies it may be reflect an early response to accommodate an overabundance of misfolded proteins found even in the cancer precursor. Additionally, the increased *ADRM1* mRNA expression with respect to disease stage and recurrence further argues that RPN13 may contribute to disease progression. However, we did not see differences in expression between intraepithelial (STIC) and invasive (HGSC) carcinomas. [30]. Recent studies have shown that of the 214 known genes mapping to chromosome 20q13, overexpression of *ADRM1* mRNA is the most highly correlated with this chromosomal amplification [30]. Further, in vitro overexpression of *ADRM1* has led to increase in proliferation and migration of transfected ovarian cancer cell line, ES2, and has been correlated to shortened survival and more rapid relapse [53]. However, the specific pathways affected by *ADRM1* overexpression have yet to be determined.

*ADRM1* mRNA overexpression is associated with chromosome 20q13 amplification [30], but was not obviously associated with concurrent protein level changes in ovarian cancer cell lines. RPN13 protein was expressed in all normal fallopian tube epithelium, STICs, and HGSC samples tested. Patient tumor tissue samples as well as ovarian cancer cell lines show relatively consistent protein levels regardless of *ADRM1* mRNA expression levels suggesting that RPN13 expression is heavily post-transcriptionally regulated.

Proteasome components exhibit coordinate regulation mediated by Nrf1 signaling in response to partial proteasome inhibition [54, 55], and we have described their upregulation in ovarian cancer in association with ubiquitin-proteasome system (UPS) stress and increased metabolic burden [56]. Taken together, this implies the upregulation of *ADRM1* mRNA in STIC and HGSC may reflect their increased UPS and metabolic burden when compared to normal fallopian tube epithelium.

The consistent RPN13 protein expression in HGSC and the lack of impact of *ADRM1* amplification upon sensitivity to RA190 suggest that RPN13 may still be used as a therapeutic drug target without major dose adjustments for *ADRM1* amplification status. Indeed, all of the ovarian cancer cell lines tested were sensitive to RA190, although the panel warrants expansion. Another factor may be a second target; RA190 appears to adduct to both RPN13 and UCH37 to inhibit proteasome function synergistically [57]. RA190 or similar compounds have promise for treatment of patients whose disease becomes resistant to proteasome inhibition by other proteasome licensed inhibitors, such as bortezomib. Initial studies have shown that RA190 is active against mouse models of ovarian cancer, ES2 and ID8, although they are also sensitive to bortezomib [41].

## Conclusions

We show that *ADRM1* mRNA overexpression is an early event in HGSC carcinogenesis. This is associated with *TP53* mutation and increased burden of misfolded proteins in carcinomas that likely renders the cancer cells particularly sensitive to RPN13 inhibitors. However, regardless of *ADRM1* amplification status the RPN13 protein levels are relatively consistent across ovarian cancer cell lines. Since *ADRM1* amplification was also not correlated with sensitivity to RA190, this suggests it can be tested against ovarian cancer regardless of increased *ADRM1* copy number.

## Methods

### Tissue specimens and cell culture

Studies on archived formalin-fixed, paraffin-embedded ovarian carcinoma tissue specimens selected from cases in the Department of Pathology at The Johns Hopkins Hospital were approved by the Johns Hopkins University Institutional Review Board. A total of 20 specimens were selected with pathological diagnoses of high grade serous carcinoma, of which 7 also exhibited STIC. One pathologist (DX) reviewed all of the slides for all cases independently to assess the staining, and STIC diagnoses were confirmed by 3 pathologists (JDS, AVY, DX). OAW42, OV2008, COV318 cells were maintained in Dulbecco’s modified Eagle medium (DMEM) with 10% fetal bovine serum, 100 U penicillin and streptomycin, 1 U non-essential amino acids, 1 mM sodium pyruvate. A2780, Kuramochi, OVCAR3 cell lines were maintained in RPMI medium supplemented with 10% fetal bovine serum, 100 U penicillin and streptomycin, 1 U non-essential amino acids, 1 mM sodium pyruvate (Gibco, Life Technologies, Grand Island NY).

### Western blots

Prior to lysate preparation, adherent cells were released with 0.05% Trypsin-EDTA (Gibco, Life Technologies, Grand Island NY), then harvested by centrifugation at 2500 × g for 4 min. The cell pellets were washed 3× with 1 mL DPBS followed by centrifugation at 2500 × g for 4 min. Pellets were lysed using 1 mL of M-PER® Mammalian Protein Extraction Reagent for ~100 μL of cells and processed as per manufacturer’s instructions (Pierce, ThermoFisher Scientific, Rockford IL). The protein concentration of cell lysates was quantified using a BCA Assay Kit (Pierce, ThermoFisher Scientific, Rockford IL). Intensity of RPN13 and actin bands was analyzed using the NCI software ImageJ (http://rsb.info.nih.gov/ij/index.html) and their ratio determined for normalization.

### Cell viability assay

Adherent cell lines were seeded at 2000 cells/well in 100 μL of culture medium in 96-well plates. Following overnight incubation, wells were treated with serially diluted drug at specified concentrations in 100 μL and incubated for 48 h. Cells were incubated for the final 2 h at 37 °C with 20 μL of 5 mg/mL tetrazolium salt XTT (Sigma-Aldrich, St. Louis MO). To visualize, plates were lightly tapped against paper towels, crystals were dissolved in 100 μL of DMSO (Sigma-Aldrich, St. Louis MO), and absorbance of each well read at 490 nm using a Benchmark Plus microplate spectrophotometer (BIO-RAD, Hercules CA).

### TaqMan^®^ qRT-PCR and copy number variation assay

Prior to lysate preparation, adherent cells were treated to 0.05% Trypsin-EDTA then centrifuged at 2500 × g for 4 min. Remaining cell pellets were washed 3× by 1 ml DPBS followed by centrifugation at 2500 × g for 4 min. For qRT-PCR, RNA was harvested using an RNeasy Mini Kit (Qiagen Sciences, Germantown MD) followed by reverse transcription by iScript Reverse Transcription Supermix (BIO-RAD, Hercules CA) per the manufacturer’s instructions. qPCR was performed on a Bio-Rad iCycler iQ™ Real-Time PCR Detection System using TaqMan® Fast Advanced Master Mix and *ADRM1* TaqMan probes (Cat. # 4351372) (Applied Biosystems, ThermoFisher Scientific, Rockford IL). Fold-change was calculated using the Livak method and normalized to reference gene *GAPDH*.

For the Copy Number Variation Assay, gDNA was harvested using a DNeasy Blood & Tissue Kit (Qiagen Sciences, Germantown MD). DNA was quantified using UV absorbance determined with a Nanodrop™. The realtime-PCR assay was run according Applied Biosystems’ TaqMan^®^ Copy Number protocol using 20 ng of DNA, TaqMan^®^ Genotyping Master Mix, and ADRM1 TaqMan^®^ Copy Number Probe (Cat. # 4400291) and was quantified using CopyCaller™ Software.

### Chromogenic in situ hybridization

Custom RNA in situ hybridization probes (Advanced Cell Diagnostics, Inc.) were prepared to detect the full-length *ADRM1* mRNA. RNAscope® assays were performed using the RNAscope® 2.0 FFPE Brown Reagent kit. Glass-mounted 4 μm sections of formalin-fixed, paraffin-embedded tissue (FFPE) were baked overnight at 65 °C, then deparaffinized in three 10 min incubations in xylene, with the last incubation in fresh xylene. Endogenous peroxidase activity was blocked for 10 min (P1) followed by antigen retrieval by both heat (P2) and protease (P3). Protease antigen retrieval (P3) was diluted 1:5 in sterile DPBS (Gibco, Life Technologies, Grand Island NY) and incubated for 30 min. *ADRM1* probe hybridization and amplifications were completed according to manufacturer’s instructions. After washing, an HRP-based amplification system was then used to detect the target probes by 10 min incubation with manufacturer supplied DAB solutions. To ensure RNA integrity and assay procedure, adjacent sections were also hybridized with a probe to the endogenous housekeeping protein peptidyl prolyl isomerase B (PPIB)*.* To control for non-specific staining, control probe against bacterial protein dihydrodipicolinate reductase (dapB) was used on adjacent slides. Quantification of slides was done using ACD supplied SpotStudio Software.

### *ADRM1* knockout cell line

Stable *ADRM1* knockout cell line was established using CRISPR/Cas9-mediated gene targeting strategy in the HCT116 Cell line. A 0.9-kb fragment upstream of exon 4 as a 5′-homologous arm and a 1.0-kb fragment downstream of exon 4 as a 3′-homologous arm in *ADRM1* gene (encoding RPN13) was amplified by PCR using genomic DNA from HCT116 cells as a template. The primers used were 5′-AACTCGAGTGAAGGGGACCACCGTGACTCCG-3′ and 5′-TTGAATTCTTGGACCCTGCCTTGAACTTCAGC-3′ for the 0.9-kb fragment, and 5′-AAGGATCCATGCTGGCCCTGGTTCTAACGATG-3′ and 5′-TTTGCGGCCGCTCCGAAGGCACTTAGCTGCTGC-3′ for the 1.0-kb fragment. A targeting vector was constructed by sequentially subcloning the 5′-arm, the puromycin resistance gene cassette, and the 3′-arm into pBluescript II in order to delete exon 4 of *ADRM1* gene. sgRNA-encoding DNA oligos, 5′-AAACTGCGATTCGGTTAGGAACTTC -3′ and 5′-CACCGAAGTTCCTAACCGAATCGCA -3′, target 39-bp downstream and of exon 4. The pair of annealed oligos was subcloned into the BbsI site of pX330 (Addgene 42,230), which expresses sgRNA and Cas9 simultaneously. The targeting vector and the pX330 plasmid encoding each sgRNA were transfected into HCT116 cells. The cells were cultured in DMEM supplemented with 10% FBS and 4 μg/ml puromycin for 10 days. Colonies were then picked up, and deficiency of Rpn13 proteins was screened by immunoblot analysis of cell lysates.

### Immunohistochemistry

RPN13 was stained by following PowerVision Poly-HRP IHC Detection system protocol (Leica Biosystem). FFPE sections were deparaffinized in xylene, followed by rehydration in graded ethanol. Antigen retrieval was performed by steaming specimens at 100 °C for 20 min in Target Retrieval Solution (Dako) and subsequently washed in Tris-buffered saline with Tween 20 (TBST, 0.05% Tween 20). Endogenous peroxidase was blocked, by treatment of slides with Dual Endogenous Enzyme-Blocking Reagent (Dako) for 5 min at room temperature. Sections were covered with 1:500 dilution of mouse monoclonal ADRM1 Antibody (F-12) supplied by Santa Cruz Biotechnology (sc-166,754) diluted with Antibody Dilution Buffer (ChemMate) and then incubated at room temperature for 45 min. Slides were then washed with TBST, followed by incubation with PowerVision Poly-HRP Anti-Mouse IgG for 30 min at room temperature. After three washes in TBST, sections were treated with DAB chromogen (3, 3 ‘-diaminobenzidine tetrahydrochloride; Sigma) for 20 min in the dark. Sections were counterstained with Mayer’s hematoxylin (Dako), dehydrated with ethanol and xylene, and mounted permanently.

## Additional files

Additional file 1: Figure S1. Validation of a highly sensitive and specific *ADRM1* Chromogenic In Situ Hybridization (CISH) assay. A) *ADRM1* mRNA levels in samples from 4 HGSC patients with matched FFPE blocks and frozen tissue were compared in parallel by RNAscope® 2.0 assay and qRT-PCR respectively. Increase in mRNA by qRT-PCT was mirrored in RNAscope® 2.0 assay validating the quantitative capabilities of the CISH assay. Images taken at 40× magnification. B) Protein lysates of same patients were probed by Western blot and found to express similar levels of RPN13 protein despite variable *ADRM1* mRNA levels. Ratio of RPN13 to loading control, ß-actin, were calculated by comparing pixel density as measured by ImageJ. (TIFF 1689 kb)

Additional file 2: Figure S2. PPIB positive control pictures for STIC. Matched FT, STIC, and HGSC samples as well as TMA HGSCs were probed for housekeeping mRNA, *PPIB,* by RNA-CISH to assess RNA integrity. (TIFF 9327 kb)

Additional file 3: Figure S3. SpotStudio Quantification of RNA-CISH. Using ACD SpotStudio Software, single cell analysis for CISH were done on all 7 matched normal, STIC, and HGSC samples. Example image shown. (TIFF 3493 kb)

Additional file 4: Figure S4. Distribution of *ADRM1* mRNA expression per cell in matched FT, STIC, and HGSC samples. RNA-CISH probed samples were analyzed using ACD SpotStudio for estimated *ADRM1* mRNA spots per cell. Distribution of high *ADRM1* expression cells are highest in STIC and HGSC regions of interest in all cases. (TIFF 1160 kb)

Additional file 5: Figure S5. Verification of anti-RPN13 antibody specificity**.** *ADRM1* knockout HCT116 cell line and its parental line were probed for RPN13 expression by both IHC and Western blot. No staining in knockout cells were seen by either assay. (TIFF 3640 kb)

Additional file 6: Figure S6. Consistent RPN13 protein expression in TMA HGSC (Part 1). TMA HGSC samples were assessed for *RPN13* mRNA and protein expression. mRNA levels varied between samples however, protein levels remained similar between samples. (TIFF 6401 kb)

Additional file 7: Figure S7. Consistent RPN13 protein expression in TMA HGSC (Part 2). This shows additional cases, stained as described in Figure S6.﻿ (TIFF 9590 kb)

## Abbreviations

CISH: Chromogenic in situ hybridization
CP: 20S Core particle
DAB: 3, 3′-diaminobenzidine tetrahydrochloride
FFPE: Formalin-fixed, paraffin embedded
HGSC: High grade serous carcinoma
MM: Multiple myeloma
PPIB: Peptidyl prolyl isomerase B
RP: 19S regulatory particle
STIC: Serous tubal intraepithelial carcinoma
TCGA: The cancer genome atlas
UPR: Unfolded protein response
UPS: Ubiquitin-proteasome system
XTT: 2,3-Bis-(2-Methoxy-4-Nitro-5-Sulfophenyl)-2*H*-Tetrazolium-5-Carboxanilide
