# Supplementary figures and images for: Early and consistent overexpression of ADRM1 in ovarian high-grade serous carcinoma
Source: J Ovarian Res. 2017 Aug 7;10:53. doi: 10.1186/s13048-017-0347-y (PMC5547474; doi:10.1186/s13048-017-0347-y)

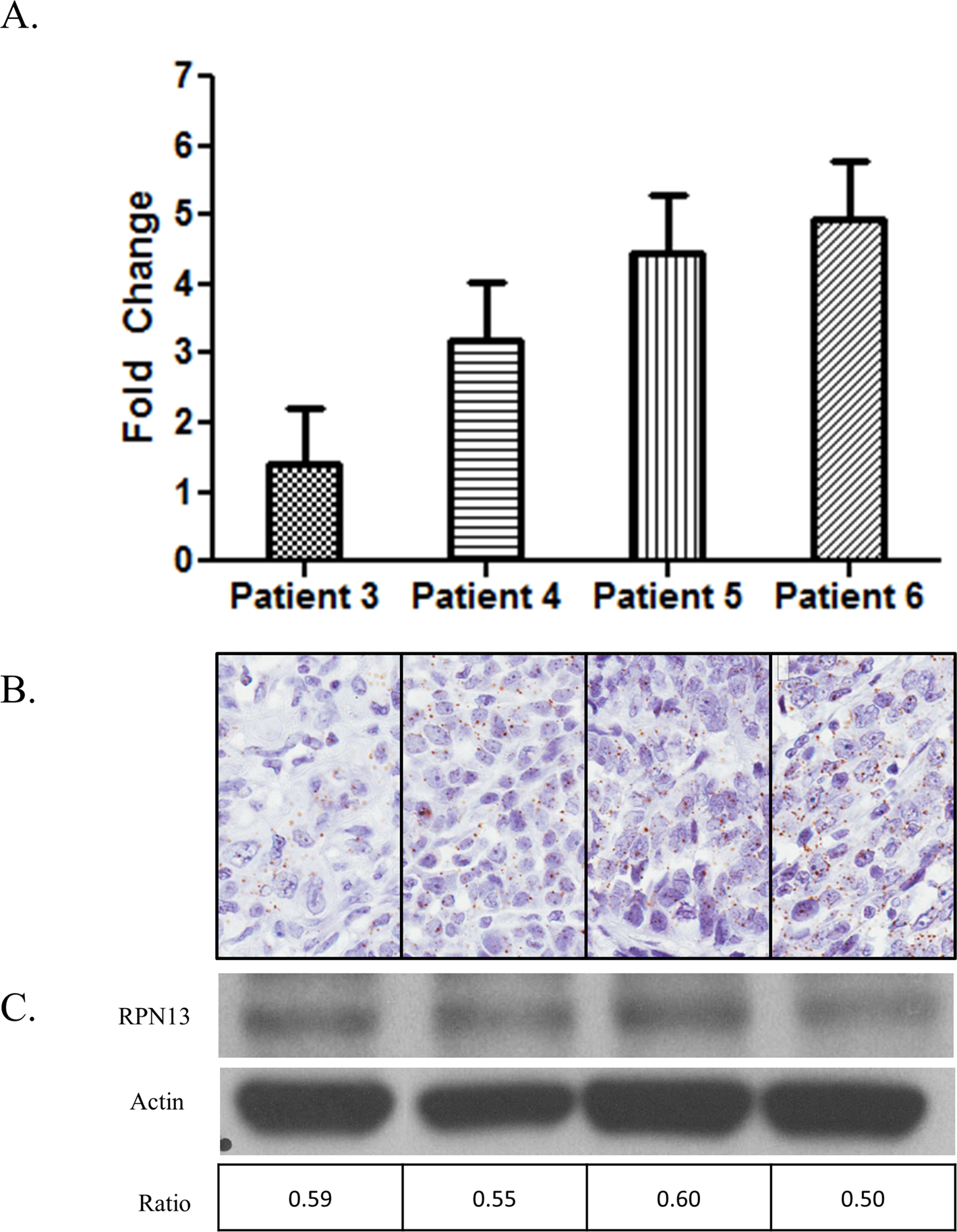

Supplement: Supplementary file 1 — Validation of a highly sensitive and specific ADRM1 Chromogenic In Situ Hybridization (CISH) assay. A) ADRM1 mRNA levels in samples from 4 HGSC patients with matched FFPE blocks and frozen tissue were compared in parallel by RNAscope® 2.0 assay and qRT-PCR respectively. Increase in mRNA by qRT-PCT was mirrored in RNAscope® 2.0 assay validating the quantitative capabilities of the CISH assay. Images taken at 40× magnification. B) Protein lysates of same patients were probed by Western blot and found to express similar levels of RPN13 protein despite variable ADRM1 mRNA levels. Ratio of RPN13 to loading control, ß-actin, were calculated by comparing pixel density as measured by ImageJ. (TIFF 1689 kb) [file 13048_2017_347_MOESM1_ESM.tif]

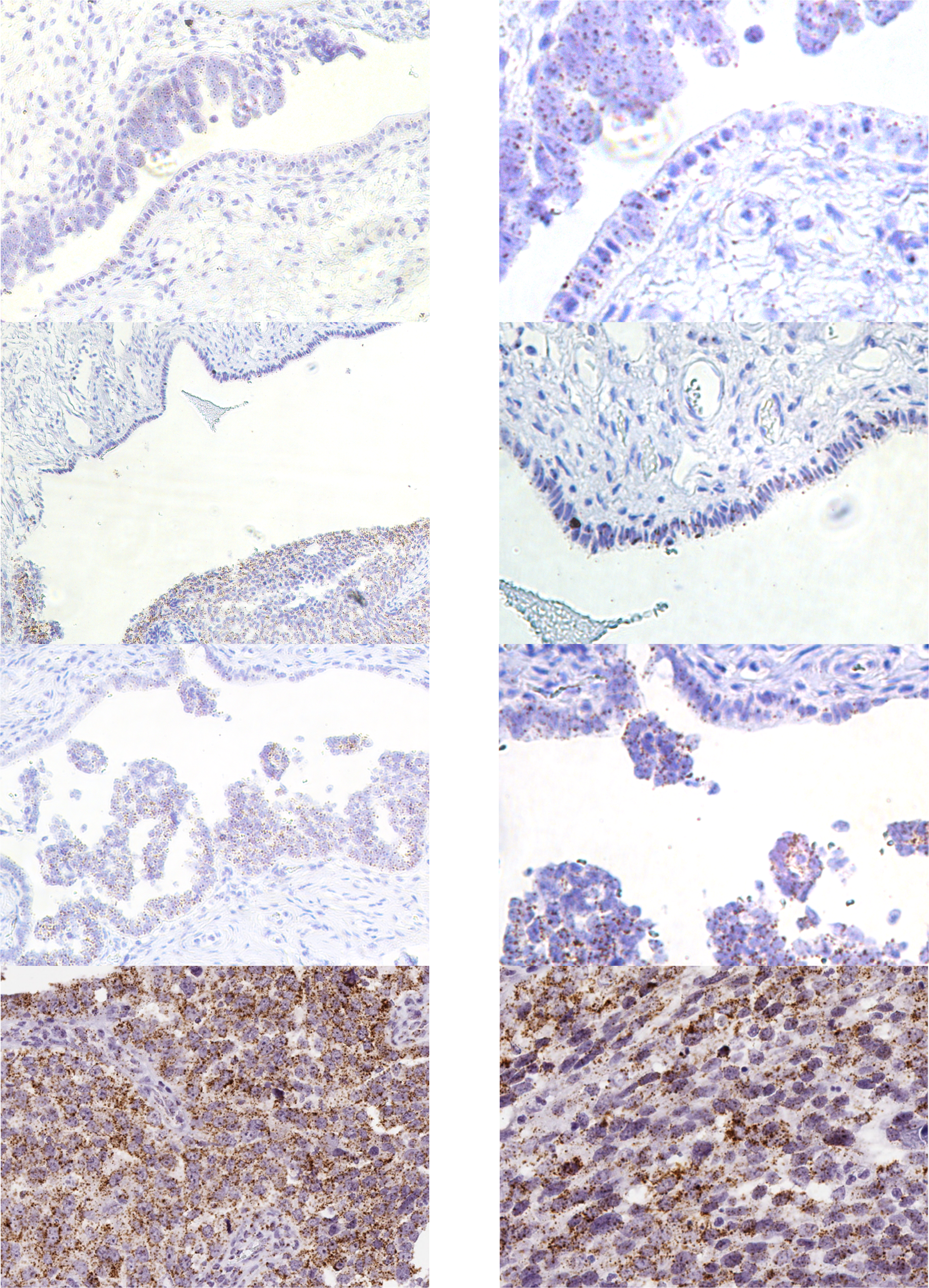

Supplement: Supplementary file 2 — PPIB positive control pictures for STIC. Matched FT, STIC, and HGSC samples as well as TMA HGSCs were probed for housekeeping mRNA, PPIB, by RNA-CISH to assess RNA integrity. (TIFF 9327 kb) [file 13048_2017_347_MOESM2_ESM.tif]

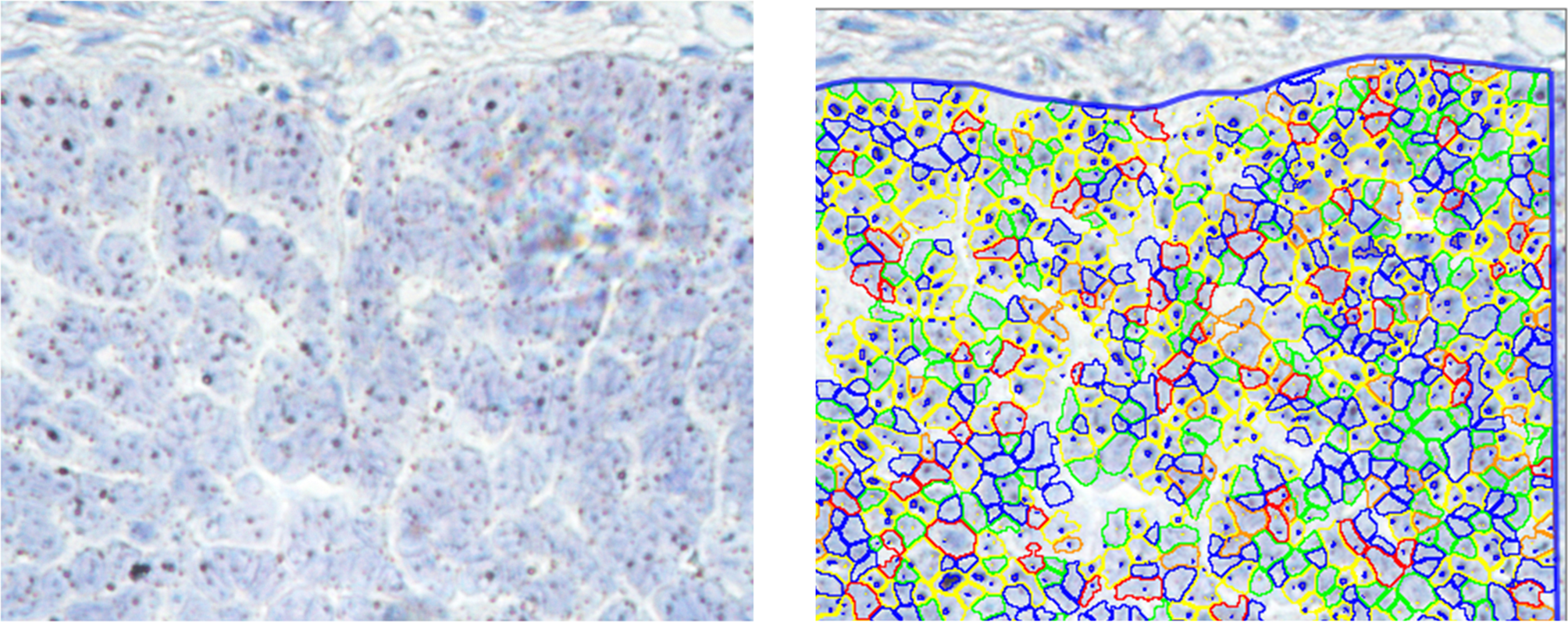

Supplement: Supplementary file 3 — SpotStudio Quantification of RNA-CISH. Using ACD SpotStudio Software, single cell analysis for CISH were done on all 7 matched normal, STIC, and HGSC samples. Example image shown. (TIFF 3493 kb) [file 13048_2017_347_MOESM3_ESM.tif]

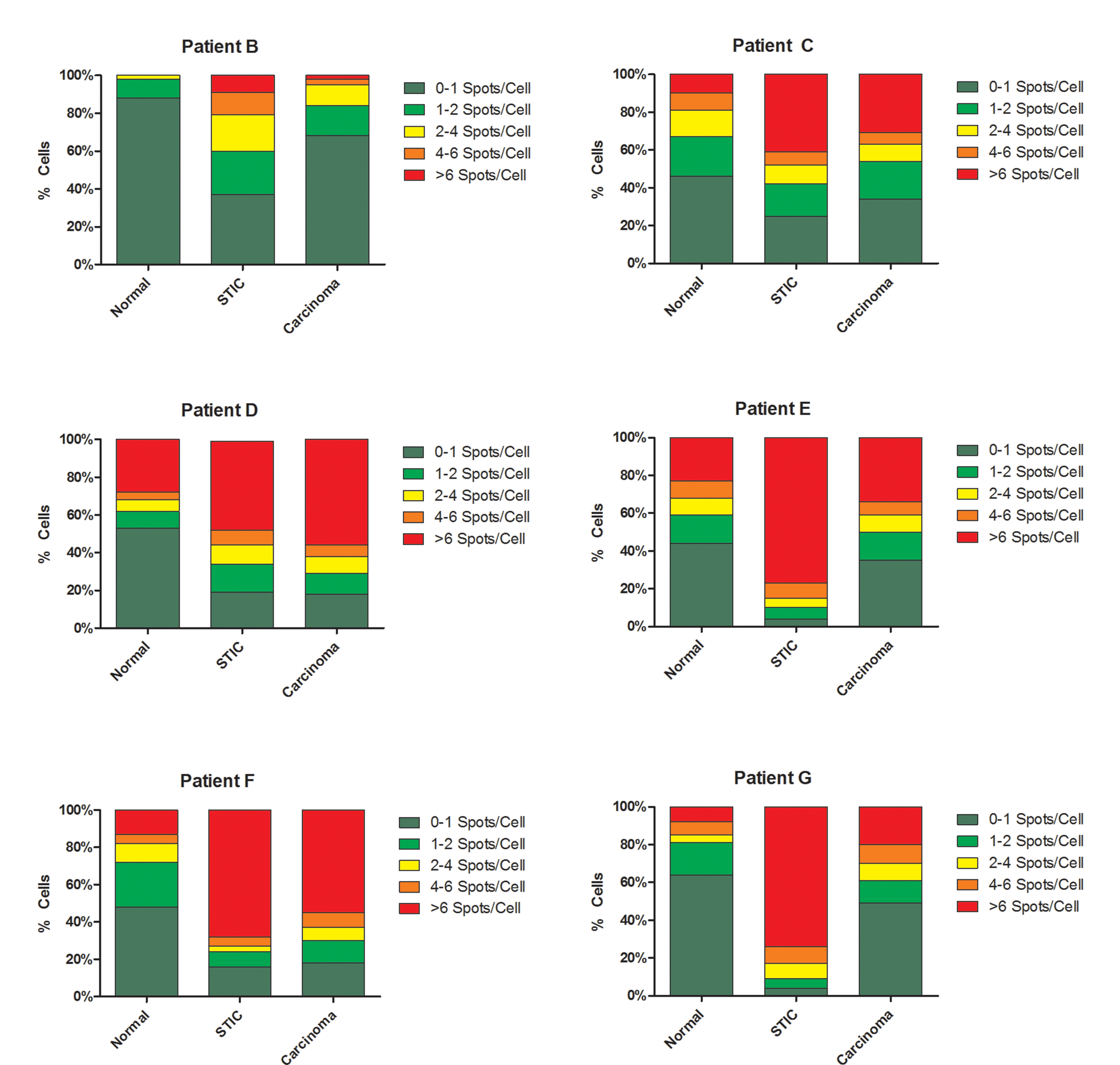

Supplement: Supplementary file 4 — Distribution of ADRM1 mRNA expression per cell in matched FT, STIC, and HGSC samples. RNA-CISH probed samples were analyzed using ACD SpotStudio for estimated ADRM1 mRNA spots per cell. Distribution of high ADRM1 expression cells are highest in STIC and HGSC regions of interest in all cases. (TIFF 1160 kb) [file 13048_2017_347_MOESM4_ESM.tif]

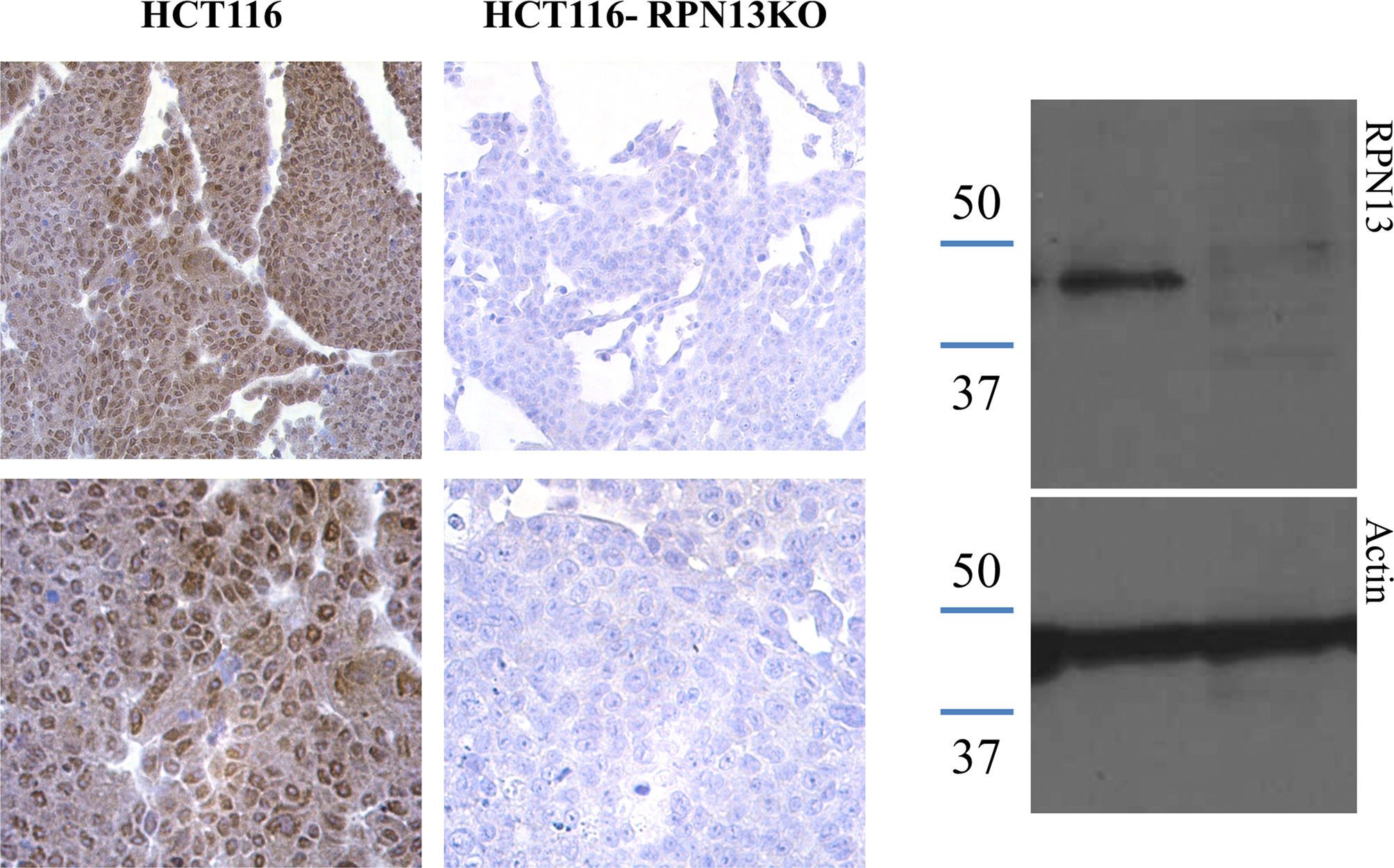

Supplement: Supplementary file 5 — Verification of anti-RPN13 antibody specificity. ADRM1 knockout HCT116 cell line and its parental line were probed for RPN13 expression by both IHC and Western blot. No staining in knockout cells were seen by either assay. (TIFF 3640 kb) [file 13048_2017_347_MOESM5_ESM.tif]

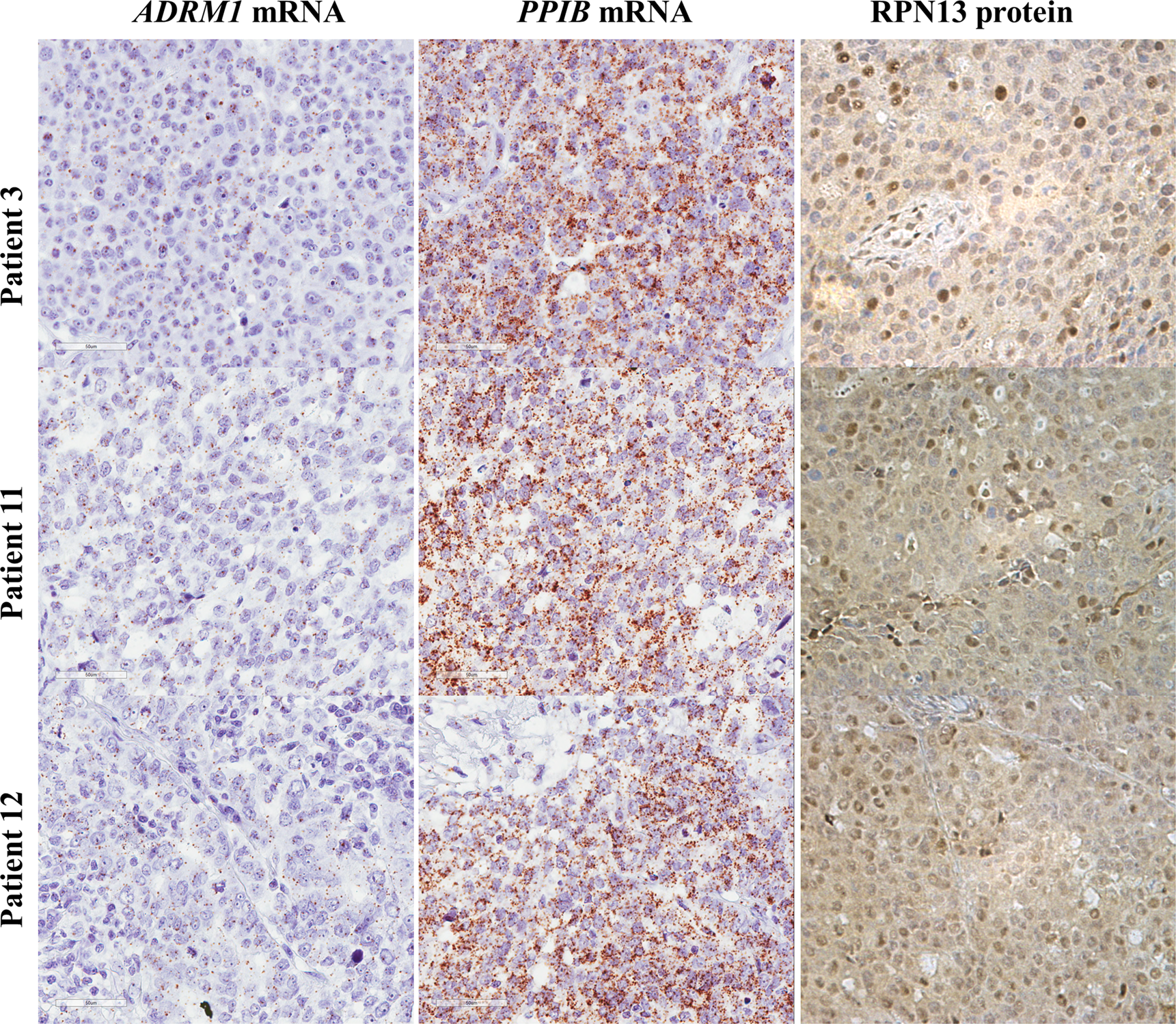

Supplement: Supplementary file 6 — Consistent RPN13 protein expression in TMA HGSC (Part 1). TMA HGSC samples were assessed for RPN13 mRNA and protein expression. mRNA levels varied between samples however, protein levels remained similar between samples. (TIFF 6401 kb) [file 13048_2017_347_MOESM6_ESM.tif]

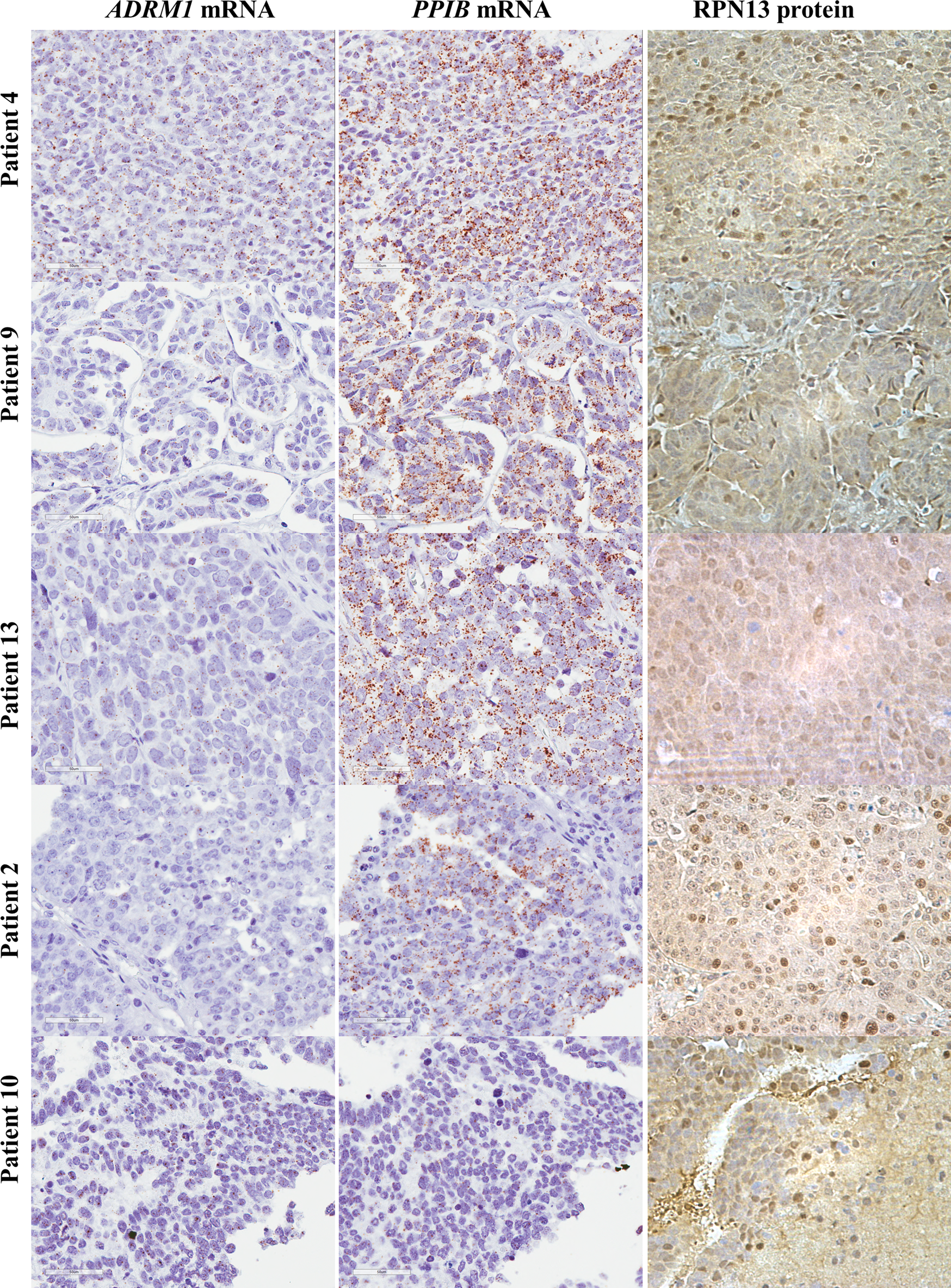

Supplement: Supplementary file 7 — Consistent RPN13 protein expression in TMA HGSC (Part 2). This shows additional cases, stained as described in Figure S6.﻿ (TIFF 9590 kb) [file 13048_2017_347_MOESM7_ESM.tif]
